# A combination of transcriptome and methylation analyses reveals embryologically-relevant candidate genes in MRKH patients

**DOI:** 10.1186/1750-1172-6-32

**Published:** 2011-05-28

**Authors:** Katharina Rall, Gianmaria Barresi, Michael Walter, Sven Poths, Karina Haebig, Karin Schaeferhoff, Birgitt Schoenfisch, Olaf Riess, Diethelm Wallwiener, Michael Bonin, Sara Brucker

**Affiliations:** 1 University Hospital Tuebingen, Department of Obstetrics and Gynecology, Tuebingen, Germany; 2 University Hospital Tuebingen, Department of Medical Genetics, Microarray Facility, Tuebingen, Germany

## Abstract

**Background:**

The Mayer-Rokitansky-Küster-Hauser (MRKH) syndrome is present in at least 1 out of 4,500 female live births and is the second most common cause for primary amenorrhea. It is characterized by vaginal and uterine aplasia in an XX individual with normal secondary characteristics. It has long been considered a sporadic anomaly, but familial clustering occurs. Several candidate genes have been studied although no single factor has yet been identified. Cases of discordant monozygotic twins suggest that the involvement of epigenetic factors is more likely.

**Methods:**

Differences in gene expression and methylation patterns of uterine tissue between eight MRKH patients and eight controls were identified using whole-genome microarray analyses. Results obtained by expression and methylation arrays were confirmed by qRT-PCR and pyrosequencing.

**Results:**

We delineated 293 differentially expressed and 194 differentially methylated genes of which nine overlap in both groups. These nine genes are mainly embryologically relevant for the development of the female genital tract.

**Conclusion:**

Our study used, for the first time, a combined whole-genome expression and methylation approach to reveal the etiology of the MRKH syndrome. The findings suggest that either deficient estrogen receptors or the ectopic expression of certain *HOXA *genes might lead to abnormal development of the female reproductive tract. *In utero *exposure to endocrine disruptors or abnormally high maternal hormone levels might cause ectopic expression or anterior transformation of *HOXA *genes. It is, however, also possible that different factors influence the anti-Mullerian hormone promoter activity during embryological development causing regression of the Müllerian ducts. Thus, our data stimulate new research directions to decipher the pathogenic basis of MRKH syndrome.

## Background

The Mayer-Rokitansky-Küster-Hauser (MRKH) syndrome (OMIM 277000) is the second most common cause of primary amenorrhea and affects at least 1 in 4,500 females. It is characterized by congenital absence of the uterus and the upper two thirds of the vagina in women with a normal female karyotype. As the ovaries are functional, women affected have physiological hormone levels and normal secondary sexual characteristics [1]. The MRKH syndrome may occur isolated (type I), or can be associated with renal or skeletal malformations, and, to a lesser extent, auditory and cardiac defects (type II) [2]. Although it is generally sporadic, familial clustering has been described, indicating a genetic cause [3]. Familial cases have been explained by autosomal dominant inheritance with incomplete penetrance and variable expressivity or by small chromosomal aberrations undetectable in standard karyotypes [4]. However, the lack of families with informative genetic histories has not allowed the identification of any locus using standard genetic linkage analysis. Investigations have therefore used a candidate gene approach based on association with other genetic diseases or involvement during embryogenesis [4].

The association of abnormalities in Müllerian duct (MD) development with renal, skeletal, cardiac and auditory defects suggests that crucial genes of fetal development and sex differentiation such as *HOX, WNT *and those encoding anti-Müllerian hormone (AMH) and its receptor are potential candidates [5,6]. The WNT genes control the production of a large family of proteins involved in intercellular signaling during embryogenesis. Heterozygous mutations of WNT4 have been detected in a subgroup of patients, but these patients also show signs of hyperandrogenism [7-10]. *HOX *genes play key roles in body patterning and organogenesis, in particular during genital tract development and the differentiation of the kidneys and skeleton. Thus, expression or function defects in one or several *HOX *genes may account for this syndrome. Furthermore, several hormones regulate physiological processes in the adult female reproductive tract by regulating *HOX *gene expression. Alterations in *HOX *gene expression that persist in the adult are a molecular mechanism by which endocrine disruptors may affect reproductive tract development [11,12]. However, structural abnormalities in *HOX *genes or in hormones regulating *HOX *expression have not been identified in women with MRKH syndrome until today [13-17]. As a third group of genes, AMH and its receptor have been regarded as causative factors in MRKH syndrome as AMH initiates MD regression in the 6th gestational week [18-22]. Mutation analyses of the *AMH *gene, however, did not support a link between MRKH syndrome and AMH yet [18,23]. Finally, mutations in other genes with a broad spectrum of activity during early development such as *WT1, PAX2 *and others have also been excluded in MRKH patients [4].

From the previous studies one can conclude that the targeted candidate gene approach has failed to decipher the causes of MRKH syndrome [2,5,24]. Recently, several recurrent copy number variants in patients with isolated and syndromic Müllerian aplasia have been described, but none of them was consistently found in a larger group of patients [25].

Cases of discordant monozygotic twins suggest that the involvement of epigenetic factors is more likely. Several studies have identified epigenetic differences, either for selected genes in monozygotic twins or in the overall epigenome [26,27].

We provide here the first study using a whole-genome approach to detect differences at the transcriptome and methylome level between MRKH patients and healthy controls. As integrated genomics becomes more and more important, the synergy between transcriptional and epigenetic gene regulation may be used to better understand the etiology of MRKH syndrome.

## Methods

### Patients

This study was approved by the ethics committee of the Eberhard-Karls-University of Tuebingen. Between July 2007 and December 2010, we had partly or completely excised 102 rudimentary uterine structures during laparoscopic-assisted neovagina in MRKH patients after informed consent was obtained [28]. As controls, we included 63 patients who underwent hysterectomy for benign disease in the same period.

Microarray analysis was performed in eight patients and eight controls to detect differentially expressed genes and seven patients and seven controls from the same group to detect differentially methylated CpG sites. Of these patients, four had MRKH type I and four had MRKH type II, including three patients with skeletal malformations, and amongst these, one with Fallot's tetralogy and one with ureter abnormalities. None of the patients had MURCS association or other complex malformations.

Analysis of serum samples at the time of surgery showed similar distribution between cycle phase one and two in the patients and control group.

Tissue samples were examined histologically before RNA and DNA were isolated. All tissue samples in both groups consisted of more than 80% myometrium.

### RNA and DNA isolation

The total RNA from myometrial pieces of rudimentary uterine tissue or normal uterus was isolated using the RNeasy^® ^Mini Kit (Qiagen, Hilden, Germany). RNA quality was checked by a Lab-on-a-Chip-System Bioanalyzer 2100 (Agilent, Boeblingen, Germany), and the concentration was determined using a BioPhotometer (Eppendorf, Hamburg, Germany). DNA was isolated using the DNeasy^® ^purification Kit (Qiagen, Hilden, Germany) according to protocol and the concentration was determined using a BioPhotometer (Eppendorf).

### Affymetrix microarray analysis

Double-stranded cDNA was synthesized from 100 ng of total RNA and subsequently linearly amplified and biotinylated using the GeneChip^® ^WT cDNA Synthesis and Amplification Kit (Affymetrix, Santa Clara, CA, USA) according to the manufacturer's instructions. 15 μg of labeled and fragmented cDNA was hybridized to GeneChip^® ^Human Gene 1.0 ST arrays (Affymetrix). Arrays were scanned using the GCS3000 Gene Chip scanner (Affymetrix) and AGCC 3.0 software. Scanned images were inspected visually to check for hybridization artifacts and proper grid alignment and analyzed with Expression Console 1.0 (Affymetrix) to generate report files for quality control.

### Quantitative real-time PCR

Relative expression of selected mRNA targets was determined by quantitative real-time PCR (qRT-PCR). 250-500 ng of total RNA was reverse transcribed using a QuantiTect Reverse Transcription Kit (Qiagen) according to the manufacturer. cDNA was diluted 1:10 before PCR amplification or preamplified using the TaqMan PreAmp Master Mix Kit (Applied Biosystems, Carlsbad, CA, USA) according to the manufacturer's protocol and diluted 1:20 for the subsequent PCR analysis. Primers were designed with Primer3 or PrimerBlast (http://biotools.umassmed.edu/bioapps/primer3_www.cgi, http://www.ncbi.nlm.nih.gov/tools/primer-blast/) and synthesized by Metabion (Metabion, Martinsried, Germany). Table 1 gives a list of PCR targets and primers.

**Table 1:** qRT-PCR targets with corresponding Affymetrix probeset ID and primer used for amplification

Target	Affymetrix cluster ID	Forward primer	Reverse primer
HOXA5	8138735	CGCCCAACCCCAGATCTA	GGCCGCCTATGTTGTCATG
HOXA9	8138749	GCTTGTGGTTCTCCTCCAGT	CCAGGGTCTGGTGTTTTGTA
PGR	7951165	TGGTGTTTGGTCTAGGATGGA	GGATCTGCCACATGGTAAGG
ESR1	8122840	GCAGGGAGAGGAGTTTGTGT	CAGGACTCGGTGGATATGG
OXTR	8085138	GCACGGTCAAGATGACTTTC	GCATGTAGATCCAGGGGTTG
PEG10	8134339	GACCCCATCCTTCCTGTCTT	GCTTCACTTCTGTGGGGATG
MFAP5	7960919	TGCTCTCGTCTTGTCTGTAAGG	ACAGGGAGGAAGTCGGAAGT
IRS1	8059470	GTTTCCAGAAGCAGCCAGAG	GGAAGATATGAGGTCCTAGTTGTGA
IRS2	7972745	CTTCTTGTCCCACCACTTGA	CAGTGCTGAGCGTCTTCTTTT
IGF2	7937772	ACACCCTCCAGTTCGTCTGT	CGGAAACAGCACTCCTCAA
WISP2	8062864	GCGACCAACTCCACGTCT	GTCTCCCCTTCCCGATACA
CDH5	7996264	ACAACGAGGGCATCATCAA	AATGACCTGGGCTCTGTTTC
SDHA	8104166	AGAAGCCCTTTGAGGAGCA	CGATTACGGGTCTATATTCCAGA
PDHB	8088384	GAGGCTGGCCACAGTTTG	GAAATTGAACGCAGGACCTT
PGRMC1	8169617	GGTGTTCGATGTGACCAAAG	TGAGGTCAGAAAGGTCATCGT
HISPPD1	8169617	TCCATCATCTGACGTTCCAC	TGGTGTTGGGAGGATCTTTG

Real-time detection of specific PCR products was performed on a LightCycler480 (Roche, Penzberg, Germany) with 5 μL of 2x QuantiTect SYBR Green PCR Kit (Qiagen). The PCR reaction was initiated by a 10 min hot start followed by 45 cycles of 95°C for 20 s, 58°C for 40 s, and 72°C for 20 s. Each PCR reaction was performed in three technical replicates. PCR efficiency was calculated from 4- or 5-fold serial dilutions of an equal mixture of all cDNAs using the following equation: E = 10^[-1/slope] [29]. To calculate the relative expression of each target, the raw Cp values were imported into qBase [30]. Three suitable reference genes (*PDH, SDHA, PGRMC1 *or *HISPPD1*) were selected according to their M-values and used for normalization of the qRT-PCR reactions [31,32].

### Illumina methylation array analysis

200-500 ng DNA was bisulfite-converted using the EZ DNA Methylation Kit (Zymo Research, Orange, CA, USA) according to the manufacturer's protocol.

Seven patient and seven control probes were evaluated for genome-wide promoter methylation using the Illumina Infinium HumanMethylation27 BeadArray.

After bisulfite conversion, each sample was whole-genome amplified (WGA) and enzymatically fragmented. The bisulfite-converted WGA-DNA samples were purified and applied to the BeadChips. Allele-specific primer annealing is followed by single-base extension using DNP- and Biotin-labeled ddNTPs. DNA methylation values, described as beta values, are recorded for each locus in each sample via BeadStudio software [33].

Differential methylation was assessed by subtracting the mean methylation level (beta value) of the patient group from the mean beta value of the reference group using BeadStudio software.

### Pyrosequencing methylation analysis

PCR and sequencing primer were designed by Qiagen using the PyroMark Assay Design Software 2.0 and are shown in Table 2. For each gene, we selected the sequence of the CpG island region which had previously been identified as differentially methylated in the array experiments. One to five adjacent CpG sites were analyzed for each CpG island. Five patient and five control samples were included in the experiment. 200 ng of isolated DNA was bisulfite-converted using the EZ DNA Methylation Kit (Zymo). 24 ng of bisulfite-treated DNA were amplified in reaction mixture containing forward and reverse primer, 1 U of HotStarTaq DNA Polymerase (Qiagen), 200 μmol each of dNTP/l and nuclease-free water. The same cycling conditions were used for all assays: denaturing at 95°C for 15 min; 35 cycles at 95°C for 10 s, at 59, 3°C for 30 s, and at 72°C for 30 s; an additional elongation step was performed at 72°C for 3 min. Gel electrophoresis was carried out on all PCR products. All PCR reactions included a no-template control and four standardized methylation controls (0%, 30%, 70%, and 100% methylated DNA). Pyrosequencing was carried out using the Pyrosequencer PSQ 96 MA (Biotage AB, Uppsala, Sweden). Results were automatically analyzed using the PSQ 96MA 1.0 software (Allele Quantification mode).

**Table 2:** Pyrosequencing targets with corresponding Illumina probeset ID and primer used for amplification

Target	Illumina Target ID	Forward primer	Reverse primer	Sequencing primer
WISP2	cg03562120	GTGTGTGTTTGGGAGTGATTT	Bio-CTCATATCCCCTACAAAACCAACTTTAA	GTTTGGGAGTGATTTTATAGTTGT
HOXA5	cg02248486	GGAATTATGATTTTTATAATTATGTAATTGGTAGTT	Bio-AACCACAAATCAAACACACATATCA	AATTATGATTTTTATAATTATGTAATTGGTAG
HOXA9	cg27009703	Bio-GTGGTGATGGTGGTGGTATAT	ACTTCAACCCCTACAACTTCCAATCCA	TCAACCCCTACAACTTCCAATCCAAAA
WT1	cg25094569	Bio-TGGATGTGATTTTGGGATAGGT	CCCATTTTTAAAACCAAACCATTTAACT	ATTTTTAAAAAATAAACAACCTTCTCTATC
GATA4	cg17795240	AAGGATTGGTTTAGGGAGAGTTTGTTTTG	Bio-TAAAATTTCACCATATTAACCAAAAACTCCTAACCTTA	GGTTTAGGGAGAGTTTGTTTTG

### Pathway analysis

Gene regulation networks were generated using Ingenuity Pathway analysis software (ingenuity^® ^systems, http://www.ingenuity.com). The dataset with differentially regulated transcripts and their corresponding expression and methylation values were uploaded into the application. The genes were overlaid onto a global molecular network developed from information in the Ingenuity Pathways Knowledge Base. Networks of these focus genes were then algorithmically generated based on their connectivity. All edges are supported by at least one reference from the literature, from a textbook, or from canonical information stored in the Ingenuity Pathways Knowledge Base. Genes from the microarray dataset that met the fold change cutoff of 1.5 and that were associated with a relevant pathway in the Ingenuity Pathways Knowledge Base were included in the analysis.

### Statistical methods

With quantitative RT-PCR normalized values were obtained from qBase. The ratio of means of patients divided by means of controls was then calculated and the log2 of this ratio is shown. For the Affymetrix microarray analysis the means of patients were divided by the means of controls and the log2 was shown as log fold change. Measurement errors were calculated using Gaussian error propagation. As usual it is assumed that normalized values obtained from qBase are lognormal distributed and a t-test (i.e. the Welch test assuming unequal variances) was applied for each gene to investigate the difference between patients and controls in qRT-PCR. The same was done for expression array data. A significance level of 5% was chosen. The percentage of methylated cytosines was obtained from pyrosequencing methylation analysis. The difference means of patients minus means of controls are shown. The same was done for the average beta values.

## Results

### Microarray expression and methylation analysis

To identify changes in the expression level of putative candidate genes, we performed microarray analysis with Affymetrix Human Gene 1.0. Analysis using the ArrayAssist 4.0 software identified 293 transcripts differentially expressed between tissue samples of MRKH patients and controls. Of these transcripts, 161 were upregulated and 132 downregulated with a fold change of at least 1.5 and a p-value of less than 0.05 (Table 3).

**Table 3:** Differential expression of genes and methylation of CpG-sites in MRKH patients compared to controls in numbers

Difference in patients from controls	n
Differential expression, total	293
Downregulated	132
Upregulated	161

Differential methylation, total	194
Hypomethylated	116
Hypermethylated	78

Overlap	9

Pathway analysis revealed genes relevant in the embryological development of the genital tract, including *HOXA *genes and hormone receptors.

The delineation of regional DNA methylation patterns has important implications for understanding why certain regions of the genome can be expressed in specific developmental contexts and how epigenetic changes might enable aberrant expression patterns and disease [27]. We therefore decided to compare whole-genome expression and methylation patterns in uterine rudiments of MRKH patients compared to control uteri. To achieve this, we performed Illumina HumanMethylation27 BeadArrays and overlaid both datasets. The analysis using the BeadStudio software identified 194 differentially methylated CpG sites in specific CpG islands. Of these sites, 78 were hypermethylated and 116 hypomethylated (Table 3).

Nine genes were detected in both datasets (*HOXA5, HOXA9, WISP2, CDH5, PEG10, MFAP5, LRRC32, RALGPS2, SMPD3*); these are termed 'overlap genes' (Table 4). CpG sites within these genes were either hypermethylated and the genes underexpressed or hypomethylated and overexpressed, except for one gene.

**Table 4:** Overlap genes: names of differentially expressed genes that contain differentially methylated CpG-sites

Probe set ID, human 1.0 genechip array	Probe set ID, 27human methylation	Gene title	Gene symbol	Fold change human 1.0 genechip array	p-value	Diff methyl 27human methylation array	p-value
8138735	cg02248486	homeobox A5	HOXA5	1.9	0.00036	-0.33	0.00015

8138749	cg26521404	homeobox A9	HOXA9	1.5	na	-0.23	0.00047

8062864	cg03562120	WNT1-inducible signaling pathway protein 2	WISP2	-1.7	0.00148	0.14	0.00096

7996264	cg22319147	cadherin 5, type 2, VE-cadherin (vascular epithelium)	CDH5	-1.6	0.00503	-0.12	0.00598

8134339	cg19107595	paternally expressed 10	PEG10	1.8	0.00290	-0.12	0.01596

7960919	cg15815843	Microfibrillar-associated protein 5	MFAP5	-2.1	0.04635	0.16	0.02451

7950555	cg20899321	Leucine-rich repeat containing 32	LRRC32	-1.6	0.00435	0.12	0.00045

7907657	cg10559803	Ral GEF with PH domain and SH3 binding motif 2	RALGPS2	2.0	0.00385	-0.11	0.00345

8002249	cg17217677	sphingomyelin phosphodiesterase 3, neutral membrane (neutral sphingomyelinase II)	SMPD3	1.6	0.00001	-0.10	0.01602

Of the nine overlap genes, six (*CDH5, MFAP5, WISP2, HOXA5, PEG10, HOXA9*) were included in the subsequent analyses and experiments as they are known to be relevant to the embryological development of the female genital tract.

### Network and pathway analysis

**Figure 1:**
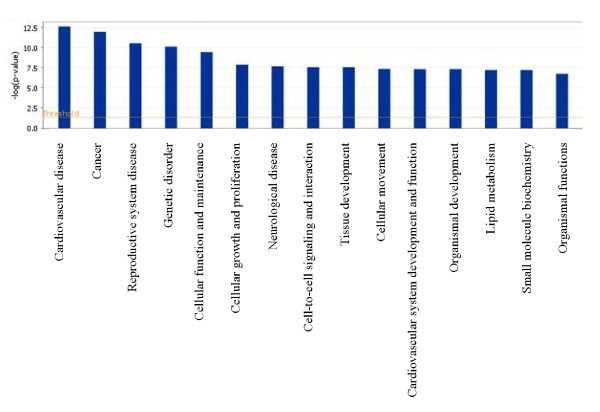
**Assignment of differentially expressed genes to functional groups**. Assignment of all differentially expressed genes to functional groups. The analysis was done with Ingenuity Pathways analysis software. Fischer's exact test was used to test for significance (shown as bars), determining the probability that each biological function assigned to the network is due to chance alone.

**Figure 2:**
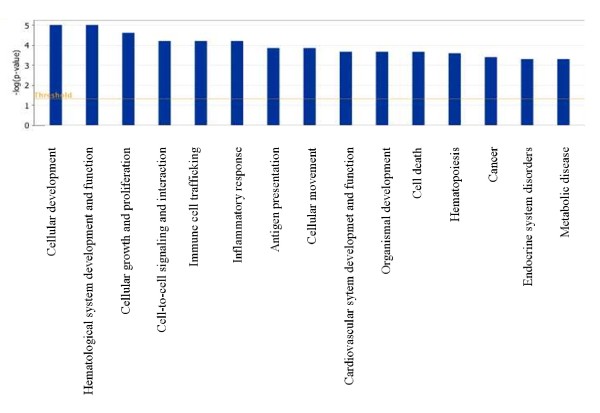
**Assignment of all genes containing differentially methylated CpG-sites to functional groups**. Fischer's exact test was used to test for significance (shown as bars), determining the probability that each biological function assigned to the network is due to chance alone.

Ingenuity Pathways analysis software (Ingenuity Systems) was used to examine the connection between the differentially methylated CpG sites and differentially expressed genes. As shown in Figures 1 and 2, differentially expressed genes and differentially methylated sites can be assigned to basic functions relevant to cell and tissue development and proliferation, cell-to-cell signaling and interaction, cellular movement, cancer, endocrine and reproductive disorders, and others (Figure 1 and 2).

**Figure 3:**
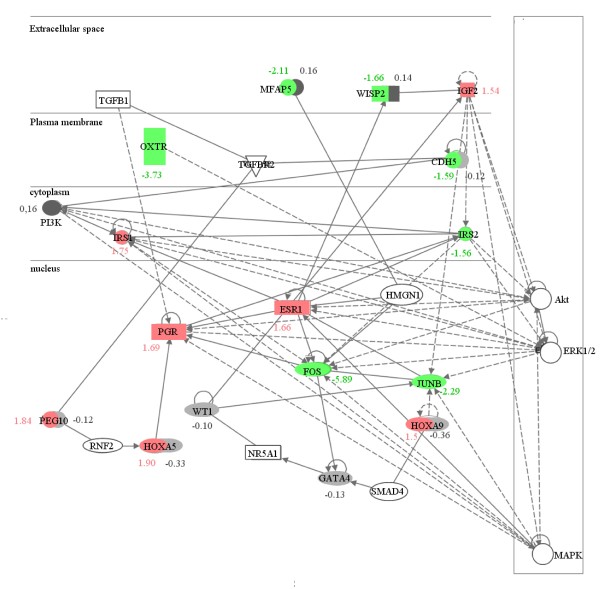
**Network of differentially expressed genes and genes containing differentially methylated CpG-sites**. The fold change of regulation in the microarray analysis, (the p-value of the significance analysis) and the percentage of differential methylation are listed below the symbols. For the purposes of simplification, only selected known gene-to-gene interactions are shown. Continuous arrow lines show direct interactions between genes and broken arrow lines show indirect interactions.

Figure 3 shows a network of differentially regulated overlap genes and other relevant genes. The network was created by fusing the expression and methylation datasets from the microarray experiments. This specific network was selected because of the known relevance of the genes included during embryological development and during functional changes of the female reproductive tract. Interactions between embryologically relevant genes, including *HOXA *genes and hormone receptors, can be clearly detected. Interacting genes are either differentially expressed, carry differentially methylated CpG sites, or both. The gene regulation network contains 15 differentially regulated genes, seven downregulated (green icons), two of these also with hypermethylated CpG sites (dark grey), and seven upregulated (red icons), three of these also with hypomethylated CpG sites (light grey). Four genes contained differentially methylated CpG sites without being differentially expressed (three hypo- and one hypermethylated). Eight genes are supplemented in the network to complete the interactions. Eleven of the transcripts shown were used for qRT-PCR validation and five for pyrosequencing.

**Figure 4:**
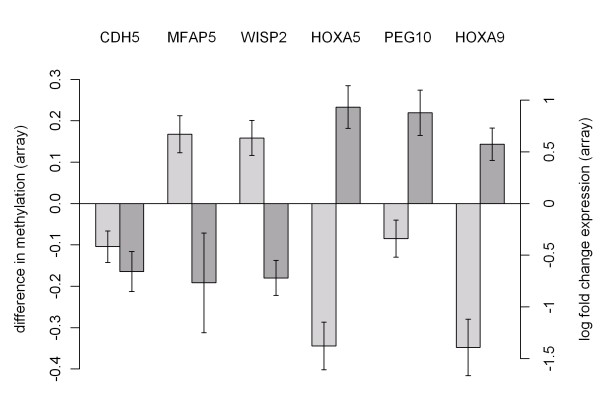
**Relation between expression and methylation in overlap genes**. Methylation (left) and expression (right) of overlap genes in MRKH patients compared to control group. Methylation is shown as difference of average beta of patients minus controls. Expression differences are shown as log fold change of patients divided by controls. Bars indicate the measurement error.

Figure 4 shows the relation between expression and methylation in overlap genes later selected for validation. CpG sites in genes were either hypomethylated with genes overexpressed or hypermethylated with genes underexpressed, except for CDH5.

### Validation of expression differences by qRT-PCR

**Figure 5:**
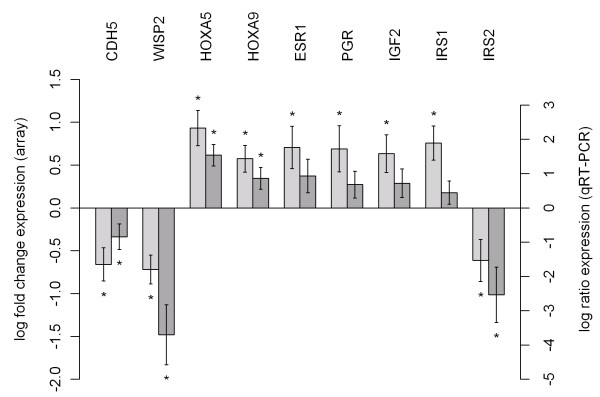
**Validation of differential expression by qRT-PCR**. Expression of overlap genes in MRKH patients compared to control group measured by array (left) and qRT-PCR (right). Array data are shown as log fold change of patients divided by controls, qRT-PCR data are shown as log ratio of patients divided by controls. Bars indicate the measurement error and stars indicate a significant difference between patients and controls (t-test, p-values <0.05).

We chose nine key players in the interaction networks for independent verification by qRT-PCR. The genes were selected because of their known relevance during embryological development. Three suitable reference genes (*PDH, SDHA, PGRMC1 *or *HISPPD1*) were selected according to their M-values and used for normalization of the qRT-PCR reactions. We were able to validate all nine genes. The results of the qRT-PCR (Figure 5) showed 100% validation efficiency in comparison to the expression data of the microarray experiment, although statistical significance was not always found.

### Validation of methylation differences by pyrosequencing

**Figure 6:**
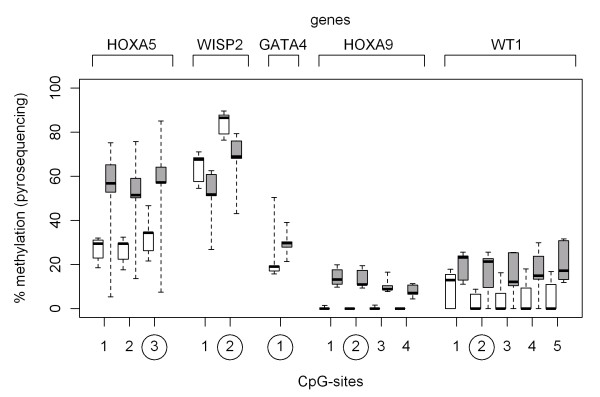
**Similar methylation in adjacent CpG sites within CpG islands**. Degree of methylation within CpG islands: shown are box-and-whisker plots of the percentage of methylated cytosines for patients (white boxes) and contols (grey boxes). The circled CpG sites correspond to the specific sites detected in the array experiments.

**Figure 7:**
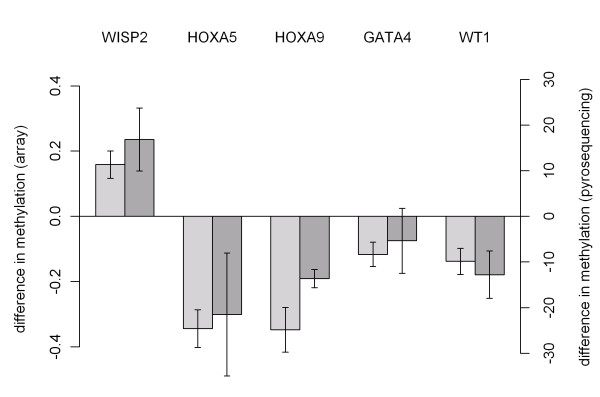
**Validation of differential methylation by pyrosequencing**. Methylation array (left) and pyrosequencing (right) of overlap genes in MRKH patients compared to control group. Both are shown as difference of patients minus controls. Bars indicate the measurement error.

Five embryologically important genes were chosen for validation of the methylation array experiments by pyrosequencing. We selected one to five CpG sites within one specific CpG island per gene for analysis (Figure 6). The CpG islands were selected according to the differential methylation in the preceding array experiments. The differential methylation status of the array experiments was confirmed for all five CpG islands within the *WISP2, HOXA5, HOXA9, GATA4 *and *WT1 *genes, thus validation efficiency was again 100% (Figure 7).

## Discussion

Although MRKH syndrome is a congenital disorder most patients are not diagnosed until puberty. Using a candidate gene approach, the underlying cause has so far not been identified [5]. Recently, a high incidence of recurrent copy number variants in patients with isolated and syndromic Müllerian aplasia has been described, but none of them was consistently found in a larger group of patients [25]. Cases of discordant monozygotic twins suggest that the involvement of epigenetic factors is more likely. The present study was the first to use a whole-genome approach to identify relevant genes, including differential expression and methylation. This allowed us to create a complex network of genes, which provided insight into possible mechanisms underlying MRKH syndrome. Our data indicate that different potential mechanisms are possible.

### Deficiency of hormone receptors

The overexpression of ESR1 and PGR in rudimentary uterine tissue from MRKH patients could be explained by a deficiency of these hormone receptors. The local overexpression may be the result of a positive feedback mechanism well known from other hormonal regulatory loops. Similar hypotheses have been postulated before but firm scientific evidence has not yet been obtained. This is the first study with an experimental setting that supports the hypothesis of locally deficient hormone receptors in MRKH syndrome.

As early as 1910, *Küster *explained the MRKH syndrome by regression of the Müllerian duct (MD) [34]. According to *Ludwig*, the MRKH syndrome results from non-fusion of the MD with the Wolffian duct (WD) [35,36]. Because the embryo is under the influence of maternal hormones, he suggested that both the non-fusion of the MD with the WD and rudimentary development of the vagina are caused by a deficiency of gestagen or estrogen receptors [35,36]. Estrogens are necessary for the embryonic development of the female reproductive tract. The special role of ESR1 in female reproductive tract development has been demonstrated by disrupting the corresponding gene in the mouse, resulting in hypoplastic uterine and vaginal tissue [37].

### Influence of estrogen on AMH and its receptor

One of the first hypotheses for the underlying cause of MRKH syndrome was an activating mutation of either the gene for the *AMH receptor *(AMHR), resulting in the inappropriate production of AMH, or the receptor itself. *Schmid-Tannwald *and *Hauser *proposed the regression of the MD due to a temporary secretion of AMH during the first fetal weeks [38]. Depending upon the amount secreted, a greater or lesser portion of the MD regressed. Nevertheless, mutation analyses of the *AMH *gene did not support a link between the MRKH syndrome and AMH at the genome level. Also, AMH protein levels in plasma and peritoneal fluid from MRKH patients were equivalent to control individuals [18,23]. Our study confirmed the data given, as we did not see any persistent differential expression or methylation patterns in the AMH or AMHR genes in adolescent MRKH patients. However, low or baseline AMH levels in a female adolescent may not necessarily be correlated with the patient's early embryonic exposure to AMH signaling [23].

It has been reported that estrogen regulates *AMH *expression [39]. The constant overexpression of ESR1 found in the rudimentary uterine tissue in our patients or the *in utero *exposure to abnormally high maternal levels of E_2 _could lead to an increasing AMH promoter activity during embryological development of the female genital tract causing uterine and vaginal aplasia.

Transcription factors involved in primary sex determination are also recruited as important regulators of AMH transcription. A common regulatory factor important for transcription of *AMH *genes is WT-1. WT-1 is essential for the embryonic development of the kidneys and gonads. GATA4 appears to play a predominant role in sex determination and sex differentiation via *AMH *gene regulation [40]. In our study CpG sites within *WT1 *and *GATA4 *were both hypomethylated compared to the control tissue. This could be a sign of stable activation leading, at least during embryological development, to activation of the *AMH *gene and thus partial regression of the MD.

### Influence of endocrine disruptors on the Müllerian duct and on the expression of HOX genes

Chemical compounds homologous to steroids can act as agonists or antagonists in fetuses exposed to them [37]. The involvement of ED with estrogen-like functions would be an explanation for the findings in MRKH syndrome, although the analyses of pregnancy histories available in all our cases so far failed to identify any clear association with drug use, illness or exposure to known substances.

Several examples of a negative impact of estrogens on uterine development are known. Transient exposure of the neonatal ewe to estrogens during critical periods specifically perturbs normal development of the uterus [41]. Estrogen inhibited caudal progression of developing MD in the turtle. It has been shown to block development of the MD when applied before the start of differentiation, and the length of the MD varied with the time point treatment was given [42].

Epithelial-mesenchymal differentiation in the murine MD is regulated by *WNT *signaling correlated with expression of *HOX *genes. Several nuclear hormonal receptors regulate the expression of multiple *HOX *genes. When a HOX gene is mutated, the body segment where it is normally expressed typically develops characteristics of the segment anterior to it, an effect known as anterior transformation. In contrast to *Drosophila*, in vertebrates, targeted mutation in a single *HOX *gene usually causes only a subtle transformation. This is because of genetic duplication and functional redundancy of adjacent genes [11]. The hypomethylation of specific CpG sites and corresponding overexpression of *HOXA9 *could be due to either exposure to a substance similar to diethylstilbestrol (DES) *in utero *or a deficient *HOXA10 *causing anterior transformation.

It is known that HOXA9 is expressed at high levels in areas destined to become the fallopian tube, HOXA10 is expressed in the developing uterus, HOXA11 is expressed in the primordia of the lower uterine segment and cervix, and that HOXA13 is expressed in the ectocervix and upper vagina. This expression pattern has been preserved in mice and humans [43]. Microarray analysis has shown organ-specific changes in gene expression profiles in the oviduct, uterus, and vagina after DES exposure. Changes in *HOX *and *WNT *expression might lead to abnormalities of segment-related positional identity in the upper part of the MD after DES exposure [43,44]. Sex steroids have been investigated in the regulation of the *HOX *genes at the 5'end of the cluster that determine posterior development, including development of the reproductive tract [45-48]. Both, *HOXA10 *and *HOXA11 *expression, is upregulated by 17ß-estradiol and progesterone. Changes in *HOX *gene expression are a potential marker for the effects of *in utero *drug use that may become apparent only at late stages of development [12]. *In utero*, DES exposure shifts *HOXA9 *expression from the oviducts to the uterus and decreases *HOXA10 *as well as *HOXA11 *expression of the uterus causing a 'T-shaped' uterus with a tube-like phenotype [47]. In human uterine and cervical cell cultures, DES has induced *HOXA9 *or *HOXA10 *gene expression [12].

Continued *HOX *gene expression in the adult has been described in the reproductive tract and may be a mechanism to retain developmental plasticity [49]. Specifically *HOXA10 *and *11 *are expressed in the endometrium and their expression varies in a menstrual cycle-dependent manner. Although no women with mutations in *HOXA10 *and *HOXA11 *have been described, patients with lower implantation rates have lower *HOXA10 *and *HOXA11 *expression in the secretory phase [43].

In addition to *HOXA9*, specific CpG sites in *HOXA5 *were hypomethylated and the gene overexpressed. This gene has a crucial role in the specification of the cervical and upper thoracic region of the skeleton. Its correct expression is important for the proper patterning of the embryo. It has been shown that ectopic *HOXA5 *expression results in abnormal differentiation [50]. In a similar manner, ectopic *HOXA5 *expression at the 5'end of the cluster might prevent normal differentiation of the MD or even regression. *HOXA5 *is known as a transcriptional regulator of multiple target genes, two of which are *p53 *and the *progesterone receptor *(PGR). The overexpression of *PGR *in patients may be induced directly by the overexpression of *HOXA5 *[51].

Finally, neonatal DES exposure is also known to cause overexpression of IRS-1 and IGF2, both of which are included in our network [52].

### Impact of WNT genes on uterine development

The *WNT *genes and products form the WNT signaling pathway which controls developmental processes. Only the *WNT4 *gene has been clearly implicated in atypical MRKH syndrome before [9]. The phenotype of *WNT9b *mutants can be rescued by activation of *WNT1 *in the WD, identifying the canonical WNT pathway as a determinant signaling process in MD elongation [37]. A recent study excluded mutations in the coding sequences of *WNT4, WNT5A, WNT7A *and *WNT9B *in 11 MRKH patients [6]. In our study, CpG sites within the *WISP2 *(WNT1 inducible signaling pathway protein 2) gene were hypermethylated and the gene underexpressed in rudimentary uterine tissue, thus the relevance is not clear yet. *WISP2 *(CCN5), a gene that is important in smooth muscle cell proliferation and migration, is an estrogen-induced gene in the uterus [53].

## Conclusion

We were able to draw important conclusions from our study, the first to compare rudimentary uterine tissue from MRKH patients and uterine tissue from healthy controls. *GATA4, WT1 *and constant overexpression of *ESR1 *might increase AMH promoter activity during embryological development, resulting in partial regression of the MD. Involvement of endocrine disruptors (ED) with estrogen-like functions might mimic this effect. The deficiency of hormone receptors may result in their overexpression and cause both the non-fusion of the MD with the WD and rudimentary development of the vagina.

The hypomethylation of specific CpG sites and the corresponding overexpression of *HOXA9 *may be due to either exposure to a substance similar to DES *in utero *or deficient *HOXA10 *causing anterior transformation. Ectopic *HOXA5 *expression at the 5'end of the cluster might prevent normal differentiation of the MD.

Using the synergetic approach of transcriptional and epigenetic regulation, our study has, for the first time, provided a deeper insight into the etiology of congenital vaginal and uterine aplasia, and has significantly advanced the explanation of MRKH syndrome. Further investigations will show which of our hypotheses is correct, but it is already clear that hormone receptors and *HOX *genes appear to play a major role and should be in focus of further examinations.

## Competing interests

The authors declare that they have no competing interests.

## Authors' contributions

KR and SB carried out the sample collection. KR drafted the manuscript. KR and GB performed the PCR and pyrosequencing experiments. MB and MW and SP carried out the microarrays and their analyses. MW, SP, KH and KS helped with the experiments. BS performed the statistical analysis. SB and MB participated in the study design and coordination and MB, OR and DW helped to draft the manuscript. All authors read and approved the final manuscript.

## Ethics Approval

This study was conducted with the approval of the Ethics Review Board of Eberhard-Karls-University, Tuebingen, Germany.

## References

[B1] Folch M Pigem I Konje JC Müllerian agenesis: etiology, diagnosis, and management Obstet Gynecol Surv 2000 55 10 644 9 10.1097/00006254-200010000-00023 11023205

[B2] Ledig S Schippert C Strick R Beckmann MW Oppelt PG Wieacker P Recurrent aberrations identified by array-CGH in patients with Mayer-Rokitansky-Küster-Hauser syndrome Fertil Steril 2011 95 5 1589 94 10.1016/j.fertnstert.2010.07.1062 20797712

[B3] Wottgen M Brucker S Renner SP Strissel PL Strick R Kellermann A Wallwiener D Beckmann MW Oppelt P Higher incidence of linked malformations in siblings of Mayer-Rokitansky-Küster-Hauser-syndrome patients Hum Reprod 2008 23 5 1226 31 10.1093/humrep/den059 18321894

[B4] Morcel K Camborieux L Programme de Recherches sur les Aplasies Müllériennes Guerrier D Mayer-Rokitansky-Küster-Hauser (MRKH) syndrome Orphanet J Rare Dis 2007 2 13 10.1186/1750-1172-2-13 17359527 PMC1832178

[B5] Sultan C Biason-Lauber A Philibert P Mayer-Rokitansky-Kuster-Hauser syndrome: recent clinical and genetic findings Gynecol Endocrinol 2009 25 1 8 11 10.1080/09513590802288291 19165657

[B6] Ravel C Lorenço D Dessolle L Mandelbaum J McElreavey K Darai E Siffroi JP Mutational analysis of the WNT gene family in women with Mayer-Rokitansky-Kuster-Hauser syndrome Fertil Steril 2009 91 4 Suppl 1604 7 19171330 10.1016/j.fertnstert.2008.12.006

[B7] Cheroki C Krepischi-Santos AC Rosenberg C Jehee FS Mingroni-Netto RC Pavanello Filho I Zanforlin Filho S Kim CA Bagnoli VR Mendonça BB Szuhai K Otto PA Report of a del22q11 in a patient with Mayer-Rokitansky-Küster-Hauser (MRKH) anomaly and exclusion of WNT-4, RAR-gamma, and RXR-alpha as major genes determining MRKH anomaly in a study of 25 affected women Am J Med Genet A 2006 140 12 1339 42 16691591 10.1002/ajmg.a.31254

[B8] Cheroki C Krepischi-Santos AC Szuhai K Brenner V Kim CA Otto PA Rosenberg C Genomic imbalances associated with mullerian aplasia J Med Genet 2008 45 4 228 32 18039948 10.1136/jmg.2007.051839

[B9] Biason-Lauber A Konrad D WNT4 and sex development Sex Dev 2008 2 4-5 210 8 10.1159/000152037 18987495

[B10] Philibert P Biason-Lauber A Rouzier R Pienkowski C Paris F Konrad D Schoenle E Sultan C Identification and functional analysis of a new WNT4 gene mutation among 28 adolescent girls with primary amenorrhea and müllerian duct abnormalities: a French collaborative study J Clin Endocrinol Metab 2008 93 3 895 900 18182450 10.1210/jc.2007-2023

[B11] Daftary GS Taylor HS Endocrine regulation of HOX genes Endocr Rev 2006 27 4 331 55 10.1210/er.2005-0018 16632680

[B12] Block K Kardana A Igarashi P Taylor HS In utero diethylstilbestrol (DES) exposure alters Hox gene expression in the developing müllerian system FASEB J 2000 14 9 1101 8 10834931 10.1096/fasebj.14.9.1101

[B13] Guerrier D Mouchel T Pasquier L Pellerin I The Mayer-Rokitansky-Küster-Hauser syndrome (congenital absence of uterus and vagina)--phenotypic manifestations and genetic approaches J Negat Results Biomed 2006 5 1 10.1186/1477-5751-5-1 16441882 PMC1368996

[B14] Mortlock DP Innis JW Mutation of HOXA13 in hand-foot-genital syndrome Nat Genet 1997 15 2 179 80 10.1038/ng0297-179 9020844

[B15] Goodman FR Bacchelli C Brady AF Brueton LA Fryns JP Mortlock DP Innis JW Holmes LB Donnenfeld AE Feingold M Beemer FA Hennekam RC Scambler PJ Novel HOXA13 mutations and the phenotypic spectrum of hand-foot-genital syndrome Am J Hum Genet 2000 67 1 197 202 10.1086/302961 10839976 PMC1287077

[B16] Burel A Mouchel T Odent S Tiker F Knebelmann B Pellerin I Guerrier D Role of HOXA7 to HOXA13 and PBX1 genes in various forms of MRKH syndrome (congenital absence of uterus and vagina) J Negat Results Biomed 2006 5 4 10.1186/1477-5751-5-4 16556301 PMC1444933

[B17] Lalwani S Wu HH Reindollar RH Gray MR HOXA10 mutations in congenital absence of uterus and vagina Fertil Steril 2008 89 2 325 30 10.1016/j.fertnstert.2007.03.033 17482600

[B18] Oppelt P Strissel PL Kellermann A Seeber S Humeny A Beckmann MW Strick R DNA sequence variations of the entire anti-Mullerian hormone (AMH) gene promoter and AMH protein expression in patients with the Mayer-Rokitansky-Kuster-Hauser syndrome Hum Reprod 2005 20 1 149 57 15550498 10.1093/humrep/deh547

[B19] Visser JA AMH signaling: from receptor to target gene Mol Cell Endocrinol 2003 211 1-2 65 73 10.1016/j.mce.2003.09.012 14656478

[B20] Guioli S Sekido R Lovell-Badge R The origin of the Mullerian duct in chick and mouse Dev Biol 2007 302 2 389 98 10.1016/j.ydbio.2006.09.046 17070514

[B21] Orvis GD Behringer RR Cellular mechanisms of Müllerian duct formation in the mouse Dev Biol 2007 306 2 493 504 10.1016/j.ydbio.2007.03.027 17467685 PMC2730733

[B22] Josso N Belville C di Clemente N Picard JY AMH and AMH receptor defects in persistent Müllerian duct syndrome Hum Reprod Update 2005 11 4 351 6 10.1093/humupd/dmi014 15878900

[B23] Resendes BL Sohn SH Stelling JR Tineo R Davis AJ Gray MR Reindollar RH Role for anti-Müllerian hormone in congenital absence of the uterus and vagina Am J Med Genet 2001 98 2 129 36 10.1002/1096-8628(20010115)98:2<129::AID-AJMG1021>3.0.CO;2-3 11223848

[B24] Bernardini L Gimelli S Gervasini C Carella M Baban A Frontino G Barbano G Divizia MT Fedele L Novelli A Béna F Lalatta F Miozzo M Dallapiccola B Recurrent microdeletion at 17q12 as a cause of Mayer-Rokitansky-Kuster-Hauser (MRKH) syndrome: two case reports Orphanet J Rare Dis 2009 4 25 10.1186/1750-1172-4-25 19889212 PMC2777856

[B25] Nik-Zainal S Strick R Storer M Huang N Rad R Willatt L Fitzgerald T Martin V Sandford R Carter NP Janecke AR Renner SP Oppelt PG Oppelt P Schulze C Brucker S Hurles M Beckmann MW Strissel PL Shaw-Smith C High incidence of recurrent copy number variants in patients with isolated and syndromic Müllerian aplasia J Med Genet 2011 10.1136/jmg.2010.082412 PMC3322361 21278390

[B26] Kaminsky ZA Tang T Wang SC Ptak C Oh GH Wong AH Feldcamp LA Virtanen C Halfvarson J Tysk C McRae AF Visscher PM Montgomery GW Gottesman II Martin NG Petronis A DNA methylation profiles in monozygotic and dizygotic twins Nat Genet 2009 41 2 240 5 10.1038/ng.286 19151718

[B27] Laird PW Principles and challenges of genome-wide DNA methylation analysis Nat Rev Genet 2010 11 3 191 203 20125086 10.1038/nrg2732

[B28] Brucker SY Gegusch M Zubke W Rall K Gauwerky JF Wallwiener D Neovagina creation in vaginal agenesis: development of a new laparoscopic Vecchietti-based procedure and optimized instruments in a prospective comparative interventional study in 101 patients Fertil Steril 2008 90 5 1940 52 10.1016/j.fertnstert.2007.08.070 18061172

[B29] Rasmussen R Meurer S, Wittwer C and Nakagawara K Quantification on the LightCycler Rapid Cycle Real-time PCR, Methods and Applications 2001 Springer Press, Heidel 21 34

[B30] Hellemans J Mortier G De Paepe A Speleman F Vandesompele J qBase relative quantification framework and software for management and automated analysis of real-time quantitative PCR data Genome Biol 2007 8 2 R19 10.1186/gb-2007-8-2-r19 17291332 PMC1852402

[B31] Goossens K Van Poucke M Van Soom A Vandesompele J Van Zeveren A Peelman LJ Selection of reference genes for quantitative real-time PCR in bovine preimplantation embryos MC Dev Biol 2005 5 27 10.1186/1471-213X-5-27 PMC1315359 16324220

[B32] Vandesompele J De Preter K Pattyn F Poppe B Van Roy N De Paepe A Speleman F Accurate normalization of real-time quantitative RT-PCR data by geometric averaging of multiple internal control genes Genome Biol 2002 3 7 RESEARCH0034 12184808 10.1186/gb-2002-3-7-research0034 PMC126239

[B33] Weisenberger D Van Den Berg D Pan F Berman B Laird P Comprehensive DNA Methylation Analysis on the Illumina^®^Infinium^® ^Assay Platform http://www.illumina.com/Documents/products/appnotes/appnote_infinium_methylation.pdf

[B34] Küster H Uterus bipartitus solidus rudimentarius cum vagina solida Z Geburtshilfe Gynakol 1910 67 692 718 13712323

[B35] Ludwig KS The Mayer-Rokitansky-Küster syndrome. An analysis of its morphology and embryology. Part I Morphology Arch Gynecol Obstet 1998 262 1-2 1 26 10.1007/s004040050224 9835997

[B36] Ludwig KS The Mayer-Rokitansky-*Küster syndrome. An a*nalysis of its m*o*rphology and embryology. Part II: Embryology Arch Gynecol Obstet 1998 262 1-2 27 42 10.1007/s004040050225 9835998

[B37] Massé J Watrin T Laurent A Deschamps S Guerrier D Pellerin I The developing female genital tract: from genetics to epigenetics Int J Dev Biol 2009 53 2-3 411 24 10.1387/ijdb.082680jm 19412895

[B38] Schmid-Tannwald I Hauser GA Atypical forms of the Mayer- Rokitansky-Kuster-syndrome Geburtshilfe Frauenheilkd 1977 37 386 392 873160

[B39] Chen G Shinka T Kinoshita K Yan HT Iwamoto T Nakahori Y Roles of estrogen receptor alpha (ER alpha) in the regulation of the human Müllerian inhibitory substance (MIS) promoter J Med Invest 2003 50 3-4 192 8 13678390

[B40] Miyamoto Y Taniguchi H Hamel F Silversides DW Viger RS A GATA4/WT1 cooperation regulates transcription of genes required for mammalian sex determination and differentiation BMC Mol Biol 2008 9 44 10.1186/1471-2199-9-44 18445271 PMC2387164

[B41] Hayashi K Carpenter KD Spencer TE Neonatal estrogen exposure disrupts uterine development in the postnatal sheep Endocrinology 2004 145 7 3247 57 10.1210/en.2004-0178 15059950

[B42] Dodd KL Wibbels T Estrogen inhibits caudal progression but stimulates proliferation of developing müllerian ducts in a turtle with temperature-dependent sex determination Comp Biochem Physiol A Mol Integr Physiol 2008 150 3 315 9 10.1016/j.cbpa.2008.04.002 18485773

[B43] Taylor HS Endocrine disruptors affect developmental programming of HOX gene expression Fertil Steril 2008 89 2 Suppl e57 8 18308065 10.1016/j.fertnstert.2007.12.030 PMC2495774

[B44] Suzuki A Urushitani H Sato T Kobayashi T Watanabe H Ohta Y Iguchi T Gene expression change in the Müllerian duct of the mouse fetus exposed to diethylstilbestrol in utero Exp Biol Med (Maywood) 2007 232 4 503 14 17392486

[B45] Cermik D Karaca M Taylor HS HOXA10 expression is repressed by progesterone in the myometrium: differential tissue-specific regulation of HOX gene expression in the reproductive tract J Clin Endocrinol Metab 2001 86 7 3387 92 10.1210/jc.86.7.3387 11443215

[B46] Ma L Benson GV Lim H Dey SK Maas RL Abdominal B (AbdB) Hoxa genes: regulation in adult uterus by estrogen and progesterone and repression in müllerian duct by the synthetic estrogen diethylstilbestrol (DES) Dev Biol 1998 197 2 141 54 10.1006/dbio.1998.8907 9630742

[B47] Du H Taylor HS Molecular regulation of mullerian development by Hox genes Ann N Y Acad Sci 2004 1034 152 65 10.1196/annals.1335.018 15731308

[B48] Taylor HS The role of HOX genes in the development and function of the female reproductive tract Semin Reprod Med 2000 18 1 81 9 10.1055/s-2000-13478 11299523

[B49] Morgan R Hox genes: a continuation of embryonic patterning? Trends Genet 2006 22 2 67 9 10.1016/j.tig.2005.11.004 16325300

[B50] Aubin J Lemieux M Tremblay M Behringer RR Jeannotte L Transcriptional interferences at the Hoxa4/Hoxa5 locus: importance of correct Hoxa5 expression for the proper specification of the axial skeleton Dev Dyn 1998 212 1 141 56 10.1002/(SICI)1097-0177(199805)212:1<141::AID-AJA13>3.0.CO;2-A 9603431

[B51] Sauter CN McDermid RL Weinberg AL Greco TL Xu X Murdoch FE Fritsch MK Differentiation of murine embryonic stem cells induces progesterone receptor gene expression Exp Cell Res 2005 311 2 251 64 10.1016/j.yexcr.2005.09.005 16223481 PMC1350973

[B52] McCampbell AS Walker CL Broaddus RR Cook JD Davies PJ Developmental reprogramming of IGF signaling and susceptibility to endometrial hyperplasia in the rat Lab Invest 2008 88 6 615 26 10.1038/labinvest.2008.29 18427555

[B53] Mason HR Lake AC Wubben JE Nowak RA Castellot JJ Jr The growth arrest-specific gene CCN5 is deficient in human leiomyomas and inhibits the proliferation and motility of cultured human uterine smooth muscle cells Mol Hum Reprod 2004 10 3 181 7 10.1093/molehr/gah028 14981145

